# Iris Arteriovenous Malformation in a Patient With Cryptogenic Cirrhosis

**DOI:** 10.1002/ccr3.72319

**Published:** 2026-04-12

**Authors:** Mehrdad Motamed Shariati, Ali Bolouki, Tayebeh Shiravi, Ali Motamed Shariati

**Affiliations:** ^1^ Eye Research Center Mashhad University of Medical Sciences Mashhad Iran

## Abstract

Iris arteriovenous malformation (AVM) is a rare vascular anomaly that may be discovered incidentally during routine ocular examination. Recognition of such ocular findings may prompt further systemic evaluation and multidisciplinary collaboration.

## 1 Case Presentation

**FIGURE 1:**
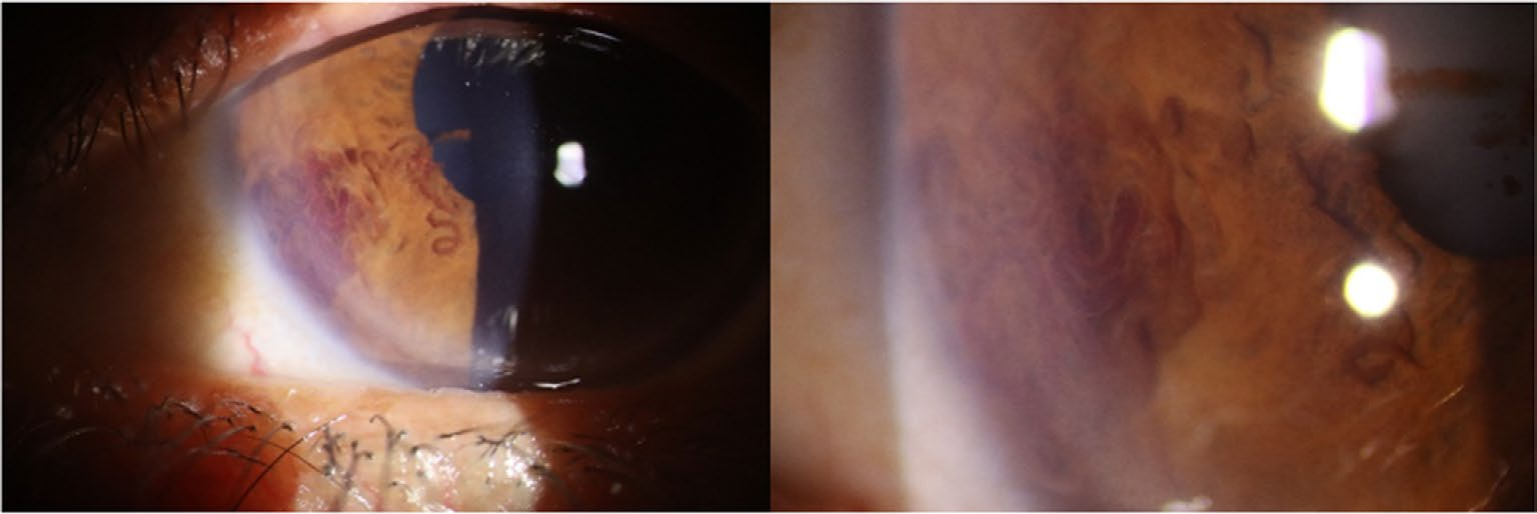
Iris vascular malformation of the right eye.

A 60‐year‐old female patient, with a known history of cryptogenic cirrhosis and esophageal varices, was referred for ocular examination as a routine check‐up. The diagnosis of cryptogenic cirrhosis was established several years prior based on clinical evaluation, laboratory findings indicating chronic liver dysfunction (elevated liver enzymes, low albumin, and prolonged prothrombin time), and imaging features of a shrunken, nodular liver with splenomegaly on ultrasonography. Extensive investigations, including viral hepatitis serologies (HBV, HCV), autoimmune liver disease panel, and metabolic workup (ceruloplasmin, α‐1 antitrypsin, and iron studies), were negative, supporting a diagnosis of cryptogenic cirrhosis. She reported no specific ocular issues. Visual acuity was 20/20 in both eyes. Intraocular pressure (IOP) measurements, obtained using Goldmann applanation tonometry, were within the normal range. Slit‐lamp examination of her left eye was unremarkable. The anterior segment examination of her right eye showed a vascular malformation consisting of dilated, tortuous vessels within the iris stroma at the inferior temporal quadrant of the iris. The vessels originating from the peripheral iris extended in an arch‐like manner from the iris root towards the pupillary margin, forming a tortuous loop near the pupil at their peak. The dilated fundus examination revealed no abnormal findings (Figure 1).

## 2 Discussion

Iris vascular abnormalities are uncommon benign lesions found within the iris stroma. These abnormalities can be categorized as iris capillary hemangiomas, iris cavernous hemangiomas, iris micro hemangiomas, iris arteriovenous malformations, and iris varices [1].

Arteriovenous (AV) malformation of the iris, synonymously called racemose hemangioma, signifies a continuity between an artery and a vein without an intervening capillary bed. Clinically, enlarged and tortuous vessels are noted within the iris stroma, originating from the iris root to the pupillary margin, forming a flexuous arch, and then back to the iris root [2].

Some (specific) iris vascular abnormalities have been observed simultaneously with other diseases, providing insights into their local or systemic causes. For instance, microhemangioma has been observed in association with myotonic dystrophy and diabetes [2]. Additionally, it has been repeatedly reported that iris capillary hemangiomas co‐occur with diffuse neonatal hemangiomatosis (DNH) or *periorbital capillary hemangiomas*. This co‐occurrence may suggest a systemic association of iris capillary hemangiomas [1].

Ultrasound biomicroscopy (UBM) and anterior segment optical coherence tomography (AS‐OCT) imaging are helpful in patients with iris vascular abnormalities. Fluorescein angiography (FA) could have provided additional diagnostic confirmation by demonstrating early arterial filling with direct venous drainage, characteristic of AVMs. However, FA was not performed in this asymptomatic patient, which remains a limitation of this case.

The presence of an iris AVM in a patient with cryptogenic cirrhosis may not be purely coincidental. Chronic liver disease is associated with systemic hemodynamic changes, including hyperdynamic circulation, increased cardiac output, and splanchnic vasodilation. These alterations can contribute to vascular remodeling and the development of portosystemic collaterals. Moreover, patients with cirrhosis frequently develop cutaneous and mucosal vascular lesions such as spider angiomas and telangiectasias, which result from increased circulating estrogen and angiogenic factors [3]. Although iris AVM is structurally distinct from these lesions, it may represent an underrecognized manifestation of systemic vascular dysregulation in cirrhosis. Syndromic associations such as hereditary hemorrhagic telangiectasia (HHT) or other congenital arteriovenous malformation syndromes were clinically excluded based on the absence of recurrent epistaxis, mucocutaneous telangiectasia, or a positive family history. Nonetheless, chronic vascular remodeling driven by endothelial dysfunction and angiogenic imbalance in cirrhosis could theoretically predispose to atypical vascular malformations, including in the iris.

## 3 Limitations

Additional imaging, such as liver CT or ultrasound images and fluorescein angiography, was unavailable for inclusion. The absence of these data limits further diagnostic and pathophysiological correlation.

## Author Contributions

**Mehrdad Motamed Shariati:** conceptualization, writing – original draft, writing – review and editing. **Ali Bolouki:** data curation, writing – original draft. **Tayebeh Shiravi:** data curation, writing – original draft. **Ali Motamed Shariati:** data curation, writing – review and editing.

## Funding

The authors have nothing to report.

## Consent

Written informed consent was obtained from the patient to publish this case report and any accompanying images. A copy of the written consent is available for review by the Editor‐in‐Chief of this journal.

## Conflicts of Interest

The authors declare no conflicts of interest.

## Data Availability

The datasets used during the current study are available from the corresponding author upon reasonable request.
